# Isolation and Characterization of Five Severe Acute Respiratory Syndrome Coronavirus 2 Strains of Different Clades and Lineages Circulating in Eastern India

**DOI:** 10.3389/fmicb.2022.856913

**Published:** 2022-06-30

**Authors:** Bharati Singh, Kiran Avula, Sanchari Chatterjee, Ankita Datey, Arup Ghosh, Saikat De, Supriya Suman Keshry, Soumyajit Ghosh, Amol Ratnakar Suryawanshi, Rupesh Dash, Shantibhusan Senapati, Tushar K. Beuria, Punit Prasad, Sunil Raghav, Rajeeb Swain, Ajay Parida, Gulam Hussain Syed, Soma Chattopadhyay

**Affiliations:** ^1^Institute of Life Sciences, Bhubaneswar, India; ^2^School of Biotechnology, Kalinga Institute of Industrial Technology, Bhubaneswar, India; ^3^Regional Centre for Biotechnology, Faridabad, India

**Keywords:** SARS CoV-2, isolation, COVID-19, growth kinetics, Indian isolates

## Abstract

The emergence of the Severe Acute Respiratory Syndrome Coronavirus 2 (SARS-CoV-2) as a serious pandemic has altered the global socioeconomic dynamics. The wide prevalence, high death counts, and rapid emergence of new variants urge for the establishment of research infrastructure to facilitate the rapid development of efficient therapeutic modalities and preventive measures. In agreement with this, SARS-CoV-2 strains were isolated from patient swab samples collected during the first COVID-19 wave in Odisha, India. The viral isolates were adapted to *in vitro* cultures and further characterized to identify strain-specific variations in viral growth characteristics. The neutralization susceptibility of viral isolates to vaccine-induced antibodies was determined using sera from individuals vaccinated in the Government-run vaccine drive in India. The major goal was to isolate and adapt SARS-CoV-2 viruses in cell culture with minimum modifications to facilitate research activities involved in the understanding of the molecular virology, host–virus interactions, drug discovery, and animal challenge models that eventually contribute toward the development of reliable therapeutics.

## Introduction

Since the emergence of the Severe Acute Respiratory Syndrome Coronavirus 2 (SARS-CoV-2) in December 2019 in Wuhan, China, the virus had an unprecedented effect on human health and wellbeing worldwide (Wu et al., 2020; Zehender et al., 2020; Zhou et al., 2020). According to WHO (2021), the virus had infected 240 million individuals globally and has so far caused 4.8 million fatalities (WHO, 2021). SARS-CoV-2 is a single-stranded, positive-sense RNA virus belonging to the genus *Coronavirus*, family *Coronaviridae*, and order *Nidovirales* (Zehender et al., 2020). The SARS-CoV-2 genome is around 30 kb in size and shares 79 and 50% homology with the genome of SARS-CoV and MERS-CoV, the causative agents of two earlier coronavirus epidemics in 2002-03 and 2012, respectively. Based on the reproductive number (R_0_), SARS-CoV-2 (2–2.2) is highly infectious than SARS-CoV (1.7–1.9) and MERS-CoV (< 1) (Petrosillo et al., 2020).

In the SARS-CoV-2 genome, ORF1a/ORF1ab encodes for two polyproteins, pp1a/pp1ab which accounts for two-thirds of the viral genome and the remaining one-third near the 3′-end encodes for four structural proteins: spike (S), envelope (E), membrane (M), and nucleocapsid (N) (Zhou et al., 2020). The spike glycoprotein is situated on the surface of the virus and plays an essential role in viral infection. It helps in receptor recognition, cell membrane fusion, and entry into the host cells. It is comprised of two subunits, S1 and S2. Furin or furin-like proteases, which are composed of multiple arginine residues, cleave the spike protein at the S1/S2 cleavage site and produce S1 and S2 subunits. Surface subunit S1 comprises a receptor-binding domain that binds to the host receptor angiotensin-converting enzyme 2. A transmembrane subunit S2 facilitates the fusion of viral and host cell membranes by making a six-helical bundle *via* the two-heptad repeat domain (Duan et al., 2020; Huang et al., 2020).

During the first wave of the pandemic, symptomatic and asymptomatic patients have coexisted together. In Pune and Madurai districts, India, 85.6 and 2.5% of patients were asymptomatic (Bhardwaj et al., 2021; Laxminarayan et al., 2021). In addition, 70.38% mortality was seen in males at a tertiary care hospital in Rishikesh, India (Tendulkar et al., 2022).

Virus isolation has not been an easy task, and hence only a few isolated strains are available with full characterization for research work. The virus was relatively easy to isolate from the samples that were collected from the throat during the first week of symptoms as it contained high viral loads, whereas the virus was difficult to isolate after the eighth day in spite of the high viral load. Virus isolation was also unsuccessful from the stool samples (Wölfel et al., 2020). In another report, attempts were made to isolate the virus from OP samples; however, only one virus was successfully isolated which had the highest viral load among all the samples (Chatterjee et al., 2021).

The whole-genome sequencing analysis was carried out in India during the initial phase of the pandemic to identify the circulating strains of SARS-CoV-2. The whole-genome sequencing data from January 2020 to July 2021 revealed that Alpha, Beta, Delta Eta, Gamma, Iota, and Kappa strains of SARS-CoV-2 contribute to 9, 1, 43, < 0.1, < 0.1, < 0.1, and 10% of the total viral isolates, respectively. The B.1.1.7 lineage was found to be predominant in the Karnataka and Maharashtra states, while in Kerala, cases belonging to clades B, I/A3i, and A2a were predominant. In Gujarat, the strains were clustered with clades 20A and 20D, whereas in Odisha, whole-genome sequencing data revealed the presence of three clades, namely, 20A, 19A, and 19B (Raghav et al., 2020; Chatterjee et al., 2021). Later on, all the other strains were replaced by delta, which became the dominant strain all over India because of its increased transmission and immune escape ability (Limaye et al., 2021). Globally, the B.1 lineage was leading. The B.1.1.7 and B.1.351 lineages were present in Qatar, while B.1.428 lineage was detected in Australia, the United States, Sweden, and Qatar. The maximum occurrence of B.1.428 sub-lineage was observed in Iraq, Tunisia, Jordan, and UAE (Benslimane et al., 2021).

The global prevalence of SARS-CoV-2 and rampant growth in the human host lead to the emergence of mutational variability among the circulating viruses. The presence of multiple variants with variability in infection/transmission and disease manifestation urges for isolation of the circulating SARS-CoV-2 variants to enhance our understanding of variant-specific differences in the viral growth characteristics, host interactions, and disease pathogenesis. In this study, five circulating strains of SARS-CoV-2 belonging to early clades have been isolated from laboratory-confirmed COVID-19 patient swab samples collected during the first COVID-19 wave in Odisha, India. The isolated strains have been further characterized and sequenced to enable the utilization of these isolates as resources in research and development toward prevention and effective therapeutic intervention against COVID-19. Due to the acquisition of several mutations in the SARS-CoV-2 strain, the efficacy and effectiveness of the currently available vaccines are also a major point of concern. Thus, further studies have been conducted to check the neutralizing capacity of sera from vaccinated persons.

## Materials and Methods

### Cells, Viruses, and Antibodies

Vero E6, Vero, BHK-21, HEK293T, and Huh7 cells were maintained in high-glucose DMEM supplemented with 10% fetal bovine serum and 1X penicillin/streptomycin. CaCo_2_ cells were maintained in DMEM supplemented with 20% fetal bovine serum and 1X penicillin/streptomycin. The THP-1 and RAW 264.7 cells were maintained in RPMI supplemented with 10% fetal bovine serum, 1X penicillin/streptomycin, 10 mM sodium pyruvate, 1M HEPES, and glucose. The details about all eight cell lines are provided in Table 1. All the cell cultures were maintained in a humidified environment with 5% CO_2_ at 37°C. SARS-CoV-2 spike and nucleocapsid antibodies were procured from Abgenex, India.

**TABLE 1:** Details of the various cell lines used in this study.

S. No	Cell line	Source
1.	Vero	Monkey kidney epithelial cell line
2.	Vero-E6	Monkey kidney epithelial cell line
3.	HEK 293T	Human embryonic kidney cell line
4.	Huh-7	Human hepatoma cell line
5.	CaCo2	Human colon epithelial cell line
6.	BHK-21	Hamster kidney epithelial cell line
7.	THP	Human monocytes cells
8.	RAW 264.7	Mouse monocytes cells

### Virus Infection

The cells were seeded a day before infection such that they attain confluency on the day of infection. Next day respective virus infection was given at a multiplicity of infection (MOI) of 0.1 in serum free media for 1.5 h at 37°C with gentle rocking for every 15 mins. After 1.5 h, the inoculum was removed, and cells were washed two times with PBS and supplemented with complete media. Five different viral strains were isolated and characterized in the current study. The details regarding these viral strains are mentioned in Table 2.

**TABLE 2:** Accession numbers of the genome sequence and clade information of the viral RNA from source swab samples (S) and isolated and culture adapted viruses (A) used in this study.

Name	Accession no	Clade
		
ILS01	EPI_ISL_463010 (S)	19A
	MW559533.2 (A)	19A
ILS02	EP_ISL_3039724 (S)	20A
	EPI_ISL_1190402 (A)	19B
ILS03	EPI_ISL_463032 (S)	20A
	EPI_ISL_1196305 (A)	20A
ILS15	EPI_ISL_463054 (S)	20B
	MW828325.1 (A)	20A
ILS24	EPI_ISL_463058 (S)	19B
	MW828330.1 (A)	19B

### Specimen Collection

Oropharyngeal (OP) swab samples from COVID-19-confirmed patients were collected in VTM from Odisha from April to June 2020. The samples were tested for the presence of virus by qRT-PCR, and samples with Ct (cycle threshold) values below 15 were subsequently used for virus isolation. Upon confirmation of infection, the samples were aliquoted and kept in deep freezers until further use.

### Ethics Statement

The current studies involving swab samples from human participants were reviewed and approved by the Institutional Human Ethics Committee, Institute of Life Sciences. The Institutional Ethics Committee (IEC)/Institutional Review Board (IRB) reference number is 96/HEC/2020. The written consent form duly signed by the participants/legal guardian was taken into consideration for this study.

### Virus Isolation

Oropharyngeal samples of confirmed COVID-19 patients were used for the isolation of the virus. The OP sample was diluted 1:1 with DMEM supplemented with antibiotics and antifungal agents and filtered through a 0.22-μm filter. Viral infection was carried out according to the method described earlier. The infected cells were regularly monitored for cytopathic effects (CPE) (Harcourt et al., 2020b). Then, 72 h post-infection (hpi), the culture supernatants were collected, and the clarified supernatants (at 3,000 rpm for 5 min) were used as inoculum for the subsequent (second) passage of virus in naïve Vero E6 cells. This process was repeated every 48 h up to the 10th passage. RNA isolated from the culture supernatants was used for the confirmation of SARS-CoV-2 virus isolation by qRT-PCR (Kumar et al., 2021). Virus titers in the culture supernatants were estimated by TCID50 assay. RNA isolated from the 10th passage virus was used for determining the whole-genome sequence. SARS-CoV-2 virus isolation and culture were conducted in the biosafety level-3 containment facility according to the guidelines issued by the Department of Biotechnology, Government of India. This study has been approved by the Institutional biosafety committee (IBSC) (IBSC file no. V-122-MISC/2007-08/01).

### Viral RNA Extraction and Estimation

RNA isolation from culture supernatant was performed using the QIAamp Viral RNA Kit (Qiagen, cat. no. 52906) according to the manufacturer’s instructions. The isolated RNA was subjected to qRT-PCR for determining the viral load by absolute quantification by real-time RT-PCR using the Takara PrimeScript*™* one-step RT-PCR Kit (RR055A) with forward (5′-GTGAAATGGTCATGTGTGGCGG-3′) and reverse (5′-CAGATGTTAAAGACACTATTAGCATA-3′) primers and probe (5′-FAM-CAGGTGGAACCTCATCAG GAGATGC-BHQ-3′) targeting the SARS-CoV-2 RdRp gene. A standard curve was generated using known quantities of SARS-CoV-2 viral RNA purified from the viral stock supernatants.

### Plaque Assay

To determine the viral titer, a plaque assay was performed as described previously (Mishra et al., 2016). In brief, 80% of confluent Vero E6 cells were infected with a serially diluted viral culture supernatant. Subsequently, the cells were overlaid with a complete methylcellulose medium and maintained in the incubator at 37°C with 5% CO_2_. After the development of the visible plaques (6–7 days), the plaques were fixed by adding 8% formaldehyde. Later on, the cells were stained using crystal violet. The number of plaques was counted as plaque-forming units/mL (PFU/mL).

### Tissue Culture Infectious Dose 50 Assay

Vero E6 cells seeded at 90% confluency in 96-well plates were infected for 1 h at 37°C with 100 μl of serially diluted (10-fold) virus inoculum in DMEM with 2% FBS. Then, at 1 hpi, the inoculum was aspirated, and the cells were replenished with fresh media. Three days post-infection, the cells were fixed in 4% paraformaldehyde and stained with 1% crystal violet to determine the cytopathic effects (CPE). The median tissue culture infectious dose (TCID50) was determined by the Reed and Muench method (Reed and Muench, 1938).

### Immunofluorescence Assay

The immunofluorescence assay was performed according to the method described by Kim et al. (2013) for the detection of infected cells. The Vero E6 cells grown on glass coverslips were infected with 0.1 MOI of respective isolates, and 48 h post-infection, the cells were fixed in 4% paraformaldehyde. Subsequently, the cells were permeabilized and blocked for 1 h with PBS containing 0.1% TritonX-100 and 3% BSA, followed by incubation with an antibody targeting the SARS-CoV-2 nucleocapsid overnight at 4°C. After washing, the cells were stained with the respective Alexa Fluor-conjugated secondary antibody (Invitrogen, Carlsbad, CA) for 1 h at room temperature. After the final wash, the coverslips were mounted onto ProLong Gold Antifade (Invitrogen, Carlsbad, CA). Images were captured under a × 100 oil immersion objective lens using a Leica TCS SP5 Confocal Microscope for the detection of protein.

### Western Blot Analysis

Immunoblot analysis was carried out as mentioned before (Kim et al., 2013). In brief, cells were lysed in RIPA buffer (20 mM Tris-HCl [pH 7.5], 150 mM NaCl, 50 mM NaF, 1 mM Na3VO4, 0.1% SDS, and 0.5% TritonX-100) containing the protease inhibitor cocktail (Thermo Fisher Scientific, Waltham, MA, United States). The whole-cell lysates (WCL) were subjected to SDS-PAGE and transferred to nitrocellulose membrane (Thermo Scientific, Waltham, MA, United States), followed by blocking and immunoblotting with antibodies specific for SARS-CoV-2 spike and nucleocapsid proteins.

### Micro-Neutralization Assay

Micro-neutralization assay was performed as mentioned before (Zhao et al., 2018). In India, initially, only two vaccines, Covaxin and Covishield, were given emergency approval and used in Government-run COVID-19 vaccination drives. The vaccinated sera from Covaxin and Covishield vaccinated healthy individuals with no history of SARS-CoV-2 were collected. Briefly, serum samples were heat-inactivated for 60 min at 56°C and filtered through a 0.22 μm syringe. These samples were then serially diluted twofold in a 96-well plate starting from 1:10 and then mixed with an equal volume of virus solution containing 1,000 TCID50 of SARS-CoV-2. This serum—virus complex was incubated for 1 h at 37°C followed by addition in duplicate to a 96-well plate containing 90% confluent Vero E6 monolayer. The plates were incubated for 36 h at 37°C in a humidified atmosphere with 5% CO_2_. Afterward, the cells were washed and fixed with 4% paraformaldehyde followed by blocking with 2% BSA for 1 h at room temperature. Cells were then incubated with SARS-CoV-2 rabbit anti-nucleocapsid antibodies for 1–2 h, followed by washing with PBS for three times and 1 h incubation with horseradish peroxidase-conjugated goat anti-rabbit IgG. After that, an equal volume of 3,3′,5,5′-tetramethylbenzidine substrate was added to each well for 15 min with the termination of the reaction by the addition of 2N H_2_SO_4_. The plates were read at 450/620 nm using a microplate reader. The neutralization percentage in each well was determined according to the formula: 100 –[(X – average of “novirus” wells)/(average of “virus only” wells – average of “novirus” wells) *100], where X is the read for each well. Non-linear regression curve fit analysis over the dilution curve was performed in the GraphPad Prism 5 software while setting the top and bottom constraints at 100 and 0%, respectively (Amanat et al., 2020; Manenti et al., 2020).

### Viral Genome Sequencing and Analysis

For the whole-genome sequencing of the isolated viruses, the viral RNA amplicon libraries were prepared using the QIAseq FX DNA Library Kit and the QIAseq SARS-CoV-2 Primer Panel (Qiagen, cat. no. 180475, cat. no. 333896) as instructed in the protocol of the manufacturer. The library was sequenced using the Illumina platform. The adapter sequence used for each sample was compatible with the Illumina NextSeq 550 instrument with 96-sample configurations (Qiaseq unique dual Y-adapter kit). The average insert length was in the 250–650 bp range. The pre-processing, alignment with viral genome, consensus sequence generation, variant calling, and phylogenetic analysis of the raw data were performed as described by Raghav et al. (2020).

### Statistical Analysis

Statistical analysis was performed using GraphPad Prism software version 5. Data were presented as mean ± standard deviation (SD). Either two-way ANOVA or Student’s *t*-test was used for the analysis. A *p*-value of < 0.05 was considered indicative of a significant difference, as mentioned in figure legends. The non-linear fit log (inhibitor) vs. response-variable slope was used to determine the percentage inhibition of virus infection due to vaccine-induced antibody-mediated neutralization.

## Results

### Characterization of Isolated Severe Acute Respiratory Syndrome Coronavirus 2 Circulating Strains

**FIGURE 1:**
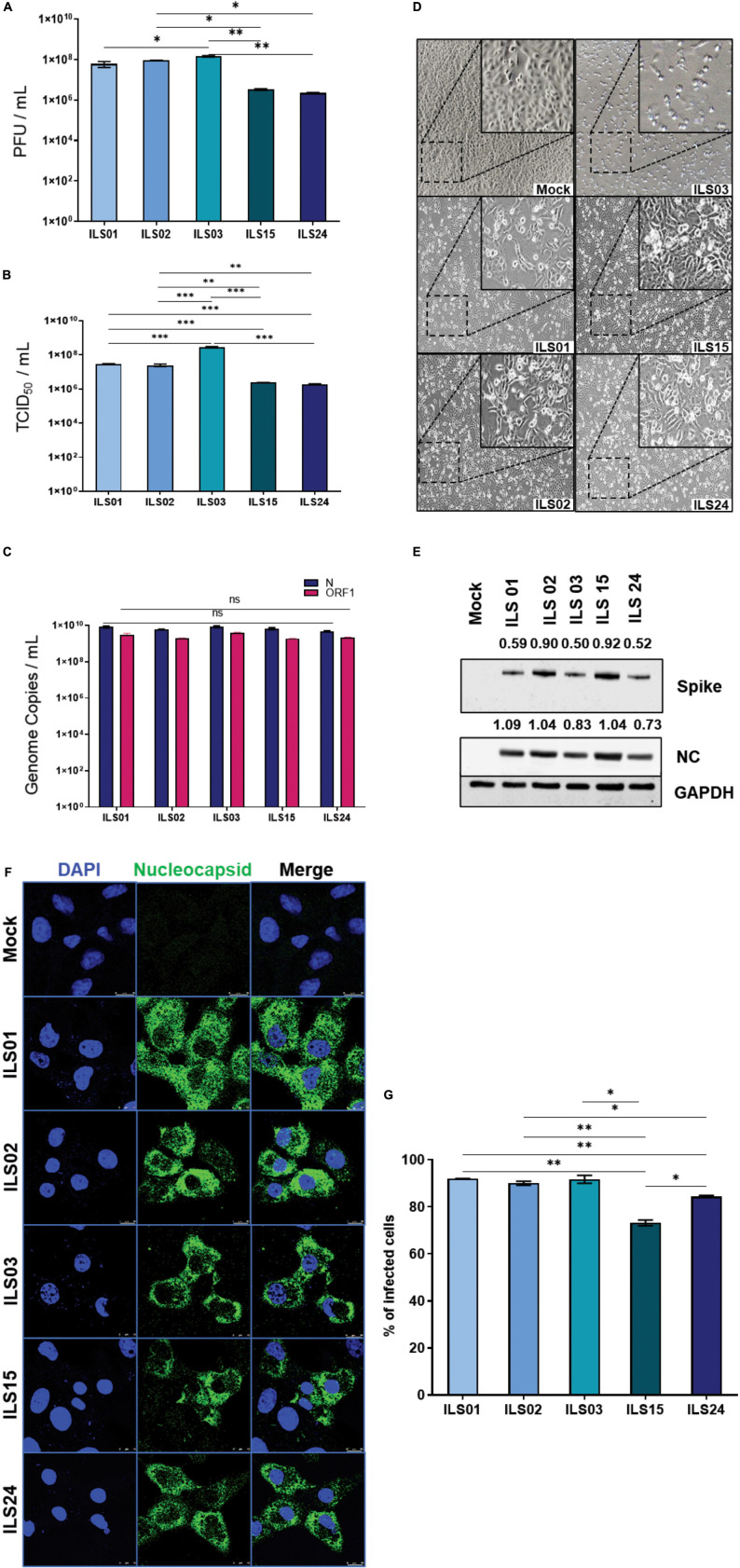
Characterization of isolated SARS-CoV-2 circulating strains. The SARS-CoV-2 circulating strains were isolated from the swab samples of COVID-19 patients via a sequential passage in Vero E6 cells, as described in the “Materials and Methods” section. The viral titers, cytopathic effects, and gene expression were determined in the 10th passage of viral stocks. Quantification of viral titers of the five isolates by plaque-forming unit (PFU) assay **(A)** and TCID50 assay **(B)**. Absolute quantification of viral genome copies in all five isolates using gene-specific primer and probes targeting SARS-CoV-2 nucleocapsid and ORF-1 gene **(C)**. Bright-field images depicting cytopathic effect in Vero E6 cells infected with the five isolates **(D)**. Western blot analysis of infected Vero E6 cell lysates with antibodies against SARS-CoV-2 spike and nucleocapsid **(E)**. GAPDH was used as a protein loading control. Quantification was done by densitometry using ImageJ software and the number represented the fold of each (spike or nucleocapsid) value normalized to the value of GAPDH. Detection of SARS-CoV-2-infected cells by immunofluorescence using an antibody against SARS-CoV-2 nucleocapsid in Vero E6 cells infected with 0.1 MOI of respective isolates **(F)** and quantification of the percentage of infection 48 hpi **(G)**. Data are the mean value ± SD (*n* = 3). Statistical significance was determined using one-way ANOVA **(A,B,G)** and two-way ANOVA **(C)** and; *P < 0.05; **P < 0.01; ***P < 0.001; ns, not significant (*P* > 0.05). Non-significant value is not shown.

There is an urgent need to isolate and establish the culture of the SARS-CoV-2 circulating viral strains to aid in research and development. Hence, attempts were made to isolate the SARS-CoV-2 virus from the OP samples of COVID-19 patients in the current study. Based on previous reports, Vero E6 cells were used for virus propagation (Harcourt et al., 2020b). The clarified supernatant collected after the 10th passage was used as viral stock for all the experiments in the current study. During the passages, RNA from the collected supernatants was subjected to qRT-PCR to confirm the presence of SARS-CoV-2. Viral titers in the supernatant of the 10th passage were determined by standard plaque (Figure 1A) and TCID_50_ assays (Figure 1B). It was observed that the viral titers of the respective isolates ranged from 10^6^ to 10^8^/mL. The Vero E6 cells were infected with 0.1 MOI of all isolates for subsequent experiments. To visualize the CPE, bright field images were captured at 48 hpi. All five isolates displayed a significant amount of virus-induced CPE; however, ILS03 displayed the highest level of CPE (Figure 1C). Absolute quantification of viral genome copies was determined by qRT-PCR, which ranged from 10^9^ to 10^10^ copies/mL (Figure 1D). Cells infected with all five isolates showed profound levels of nucleocapsid and spike proteins, as adjudged by Western blot analysis (Figure 1E). All the isolates displayed reticular cytoplasmic staining across the entire cytoplasm (Figure 1F). The quantification of the percentage of infected cells showed that around 70–90% of cells were infected at 48 hpi with the respective isolates using 0.1 MOI (Figure 1G).

### Time Kinetics of Viral Gene(s) Expression

**FIGURE 2:**
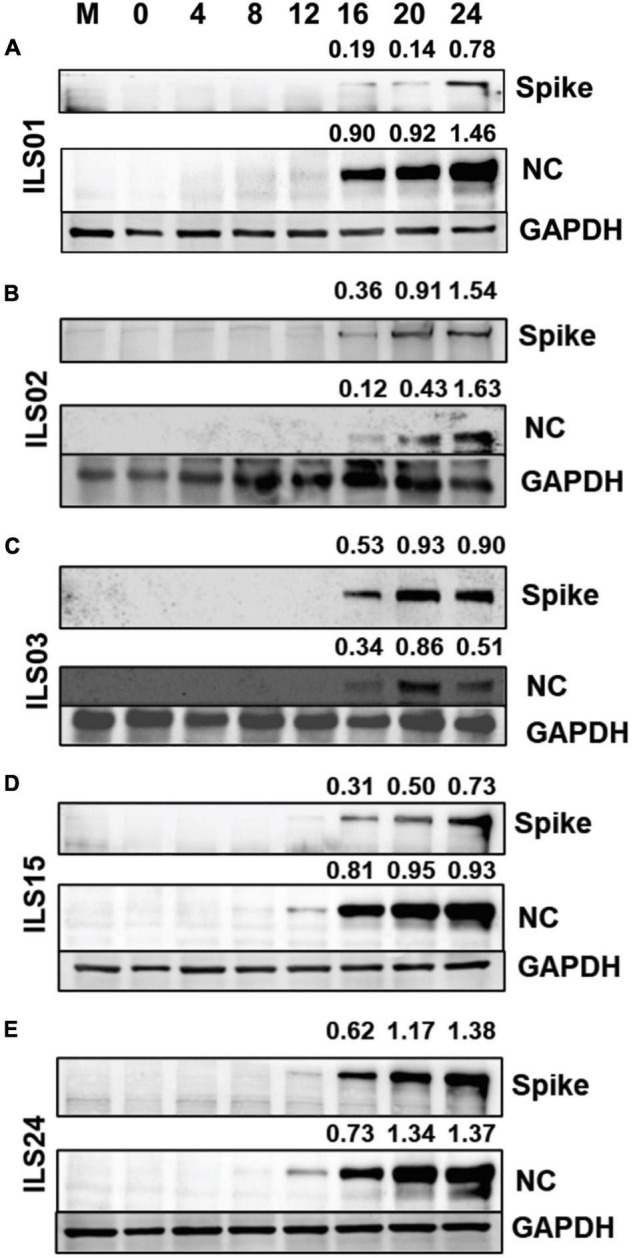
Time kinetics of viral gene(s) expression. Vero E6 cells infected with respective isolates of SARS-CoV-2 were collected at indicated time points post-infection. Cell lysates were subjected to Western blot analysis with antibodies against SARS-CoV-2 spike and nucleocapsid proteins for each isolates individually **(A–E)**. GAPDH was used as an internal loading control. Quantification was done by densitometry using ImageJ software and the number represented the fold of each (spike or nucleocapsid) value normalized to the value of GAPDH.

To access the relative differences in the kinetics of viral gene expression, a time kinetics experiment was conducted by infecting Vero E6 cells at 0.1 MOI and collecting cells at every 4-h interval for 24 h. Western blot analysis of the cell lysates for the spike and nucleocapsid proteins was evaluated. It was found that in isolates ILS01, ILS02, and ILS03, the nucleocapsid protein was observed from 16 hpi, whereas in isolates ILS15 and ILS24, it appeared from 12 hpi onward (Figure 2). Interestingly, the spike protein level was evident only after 16 hpi in all five isolates (Figure 2).

### Viral Growth Kinetics and Cytopathy

**FIGURE 3:**
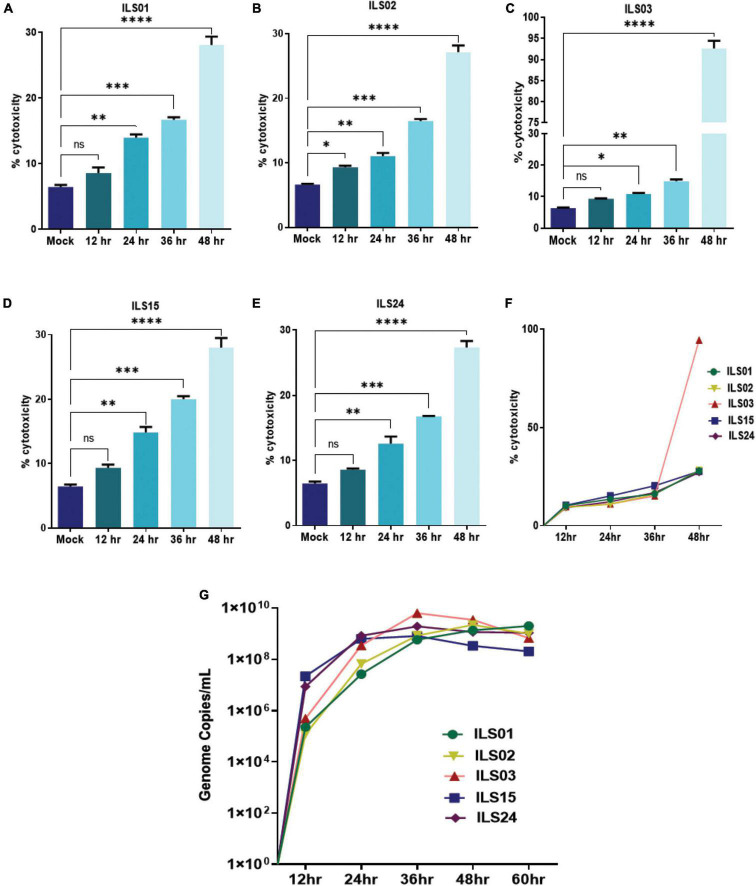
Viral growth kinetics and cytopathy. Infection-associated cytopathy was determined by calculating LDH release, as described in the “Materials and Methods” section. **(A–E)** Graph depicting the percentage of cytotoxicity in the infected Vero E6 cells at respective time points post-infection. **(F)** Line plot depicting% cytotoxicity in between the isolation. **(G)** Line plot showing a time-dependent increase in the viral genome copies in culture supernatants determined by absolute quantification of the viral genome. Data are the mean value ± SD (*n* = 3). Statistical significance was determined using one-way ANOVA; *P < 0.05; **P < 0.01; ***P < 0.001; ****P < 0.0001 ns, not significant (*P* > 0.05).

To further access the specific variations between the isolates in virus-mediated cytotoxicity and viral replication kinetics, Vero E6 cells were infected at 0.1 MOI with respective isolates, and the cell culture supernatants were collected every 12 h up to 60 hpi. Based on the LDH levels in the supernatants, it appeared that 30% cytotoxicity was induced for ILS01, ILS02, ILS15, and ILS24 isolates, whereas 90% cytotoxicity was induced for the isolate ILS03 with respect to mock at 48 hpi (Figures 3A–F). The quantification of viral genome copies in the culture supernatants suggests a steady increase in the genome copies from 12 to 36 hpi, indicating that there is an exponential increase in the release of viral particles up to 36 hpi followed by a plateau (Figure 3G).

### Susceptibility of Various Cell Lines to the Severe Acute Respiratory Syndrome Coronavirus 2 Isolates

**FIGURE 4:**
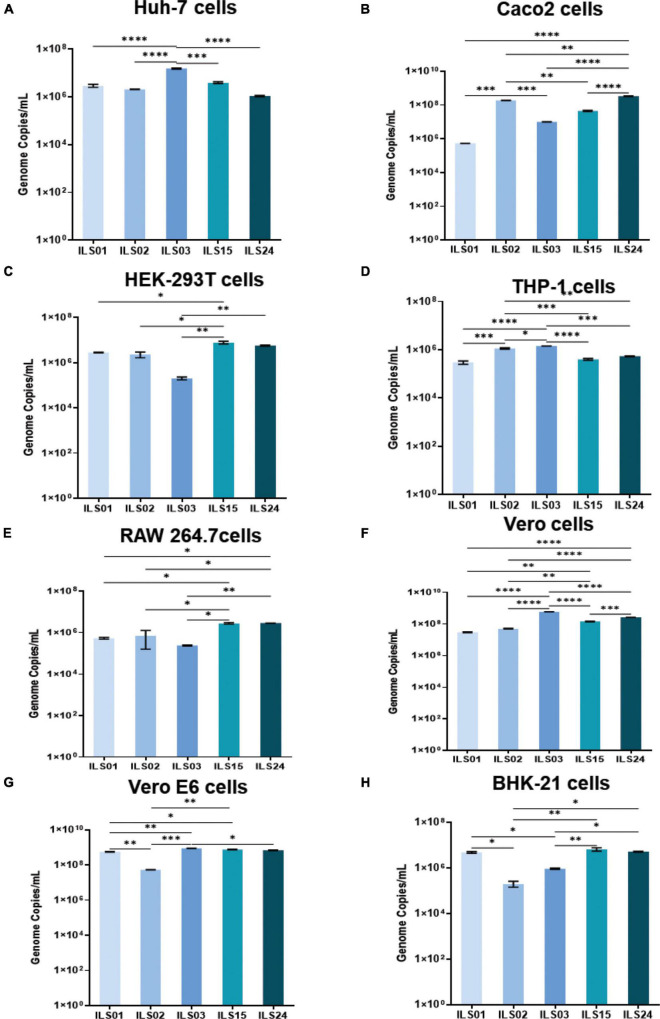
Susceptibility of various cell lines to the SARS-CoV-2 isolates. Different cell lines were subjected to infection with 0.1 MOI of respective isolates. At 24 hpi, the viral load in the culture supernatants was determined by absolute quantification of viral genome copies. Graphs depicting the viral copies per milliliter of the supernatant in Huh7 **(A)**, Caco_2_ **(B)**, HEK 293T **(C)**, THP1 **(D)**, RAW 264.7 **(E)**, Vero **(F)**, Vero E6 **(G)**, and BHK-21 **(H)** cells. Data are the mean value ± SD (*n* = 3). Statistical significance was determined using one-way ANOVA; *P < 0.05; **P < 0.01; ***P < 0.001; ****P < 0.0001 ns, not significant (*P* > 0.05). Non-significant value is not shown.

Similar to previous studies, different cell lines were used to check the susceptibility of SARS-CoV-2 isolates in this study (Caccuri et al., 2020; Harcourt et al., 2020b; Wurtz et al., 2021). Various cell lines were infected with the respective isolates, and the culture supernatants were collected at 24 hpi. The human hepatoma cell line (Huh-7), which is highly susceptible to dengue, chikungunya, and hepatitis C viruses (HCV), was found to be more or less equally susceptible to all five isolates (Figure 4A). Similarly, the CaCo_2_ cell line, which is a human intestinal epithelial cell line that has been shown by various groups to be permissive to SARS-CoV-2, was also found to be susceptible to all five isolates (Figure 4B). However, isolate ILS01 was found to be less infectious compared to the other isolates. Similarly, HEK-293T cells (a human kidney cell line) were found to be more permissive to isolates ILS01, ILS02, ILS15, and ILS 24 when compared to isolate ILS03 (Figure 4C). Immune cells predominantly show selective susceptibility to viruses. Surprisingly, it was found that the human monocyte cells THP-1 and murine macrophage cell line RAW 264.7 were permissive to all the SARS-CoV-2 isolates (Figures 4D,E). Vero E6, Vero, and BHK-21 cell lines, which are commonly used for virus propagation, were found to be equally susceptible to all five isolates (Figures 4F–H).

### Neutralization Potential of Sera Obtained From Vaccinated Individuals

**FIGURE 5:**
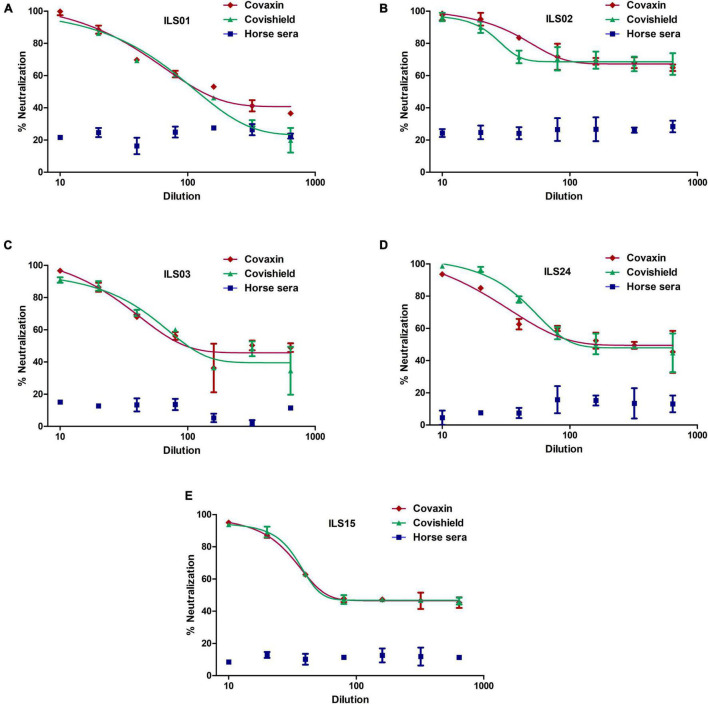
Neutralization potential of sera obtained from vaccinated individuals: The respective isolates were subjected to micro-neutralization assay using the sera obtained from Covaxin and Covishield vaccinated individuals to determine the neutralization potential of the post-vaccination sera against the respective isolates. The dose-response curves were fitted using a non-linear regression model using the GraphPad software Prism 5. **(A–E)** Neutralization efficiency of the respective vaccinated sera against the five isolates. Horse sera were used as a negative control.

To decipher any clade-specific variations toward neutralization, the neutralization capacity and protection of the vaccine-induced antibodies against the respective isolates were determined. The sera were collected 15 days post the second vaccine dose. Horse sera were used as a negative control, as it was difficult to obtain age-matched healthy control sera from individuals who had not been vaccinated or exposed to COVID-19. The micro-neutralization assay suggested that both vaccines were equally effective against all the isolates. Nearly 100% neutralization was observed at 1:10 dilution of sera obtained from vaccinated individuals, which declined to ∼50% or lower at dilution 1:160 or higher in isolates ILS01, ILS03, ILS15, and ILS24 (Figures 5A,C–E). Interestingly, infection with isolate ILS02 was neutralized even at dilution 1:160 and higher, which may be due to non-specific neutralization from the serum *per se* rather than the effect of neutralizing antibodies developed post-vaccination (Figure 5B).

### Mutation Plot of the Isolates and Source Swab Samples

**FIGURE 6:**
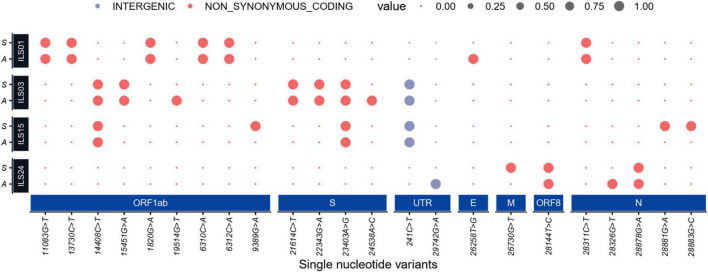
Mutation plot of the isolates and source swab samples: Dot plot representing high-quality single-nucleotide non-synonymous and intergenic variants (SNV) present in the initial viral RNA isolated from patients’ swab samples (denoted as **S**) and viral RNA from culture adapted isolates (denoted as **A**). The large dot represents the presence of an SNV in the represented sample colored by their functional annotations (gray for intergenic and red for non-synonymous SNVs). The synonymous mutation is not shown in the dot plot.

The whole-genome sequencing of culture-adapted viral isolates and viral genomes from the OP samples of patients suggested that repetitive passaging of the SARS-CoV-2 virus in Vero E6 cells did not lead to the emergence of many mutations during the adaptation in cell culture. Next, strain nomenclature and the number of mutations found in the source swab samples and isolated viruses in comparison to the Wuhan reference strain are presented in Table 3. Comparative analysis of common and unique mutations between the source samples and isolates (Table 4) and mutational plot analysis of non-synonymous mutations (Figure 6) suggest that during the culture adaptation, very minimal changes occurred. Further classification of the parent and adapted viral strains using the PANGOLIN lineage analysis suggested that all the culture-adapted viruses have the same sub-lineage as the parent strain in the clinical sample except ILS24 (the isolate is classified as A.7, whereas the parent strain as A). The isolate ILS15 (B.1.36.8) did change from the parent strains (B.1.1); however, both of them are classified as the B.1 sub-lineage (Table 5; O’Toole et al., 2021). The ILS01 isolate (clade19A) gained only one mutation (A23014C) in the spike gene during cell culture adaptation, while it retained all other 10 mutations found in the source swab samples. The isolate ILS24 (clade 19B) gained three mutations (C2143T, C10138T, and C10702T) in the ORF1ab and one mutation (G28326T) in the N genes during adaptation. It retained five mutations found in the source swab sample and gained one reversion (G26730T) to the Wuhan reference strain in the M gene. ILS03 isolated from the swab sample of clade 20A retained nine mutations found in the swab sample and gained one mutation each in ORF1ab (G19514T) and S (A24538C) genes during adaptation. Interestingly, during isolation and adaptation of ILS15 from a swab sample of clade 20B, five reversions occurred, which includes two (C8917T and G9389A) in ORF1ab and three (G28882A, G28881A, and G28883C) in N gene resulting in the reclassification of the cell culture adapted strain ILS15 in clade 20A.

**TABLE 3:** Tabular representation of genome sequences of all five isolates with reference to Wuhan strain (NC_045512).

Sample	Name	Clade	Total coverage	Missing region	Number of missing bases	Total mutation*
Swab (S)	ILS01	19A	29,665	1–30	30	11
	ILS02	20A	29,836	1–54,521–530	64	27
	ILS03	20A	29,688	1–2	2	9
	ILS15	20B	29,680	1–33	33	9
	ILS24	19B	29,805	1–30	30	6
Adapted virus (A)	ILS01	19A	29,836	1–32	32	12
	ILS02	19B	29,873	1–4	4	11
	ILS03	20A	29,836	1–4	4	11
	ILS15	20A	29,836	1–29	29	6
	ILS24	19B	29,836	1–29	29	9

*Represents intergenic, non-synonymous, and synonymous mutations in all the isolates.

**TABLE 4:** Tabular representation of SARS-CoV-2 gene-specific non-synonymous mutations in both the swab samples and cell culture adapted strains.

Name	(Number of mutation) source sample	(Number of mutation) adapted virus	Common mutations*	Reversion of mutation*	Gain of mutation*
ILS01	11	12	ORF1ab; (G11083T, C13730T, C19524T, G1820A, C6310A, C1498T, C6312A, C9451T) Spike; (C23929T), Membrane; (T26861C), Nucleocapsid; (C28311T)	None	Spike; (A23014C)
ILS03	9	11	5′-UTR; C241T, ORF1ab; (C3037T, C14408T, T20874A, C21297A), Spike; (C21614T, G22343A, A23403G)	None	ORF1ab; (G19514T), Spike; (A24538C)
ILS15	9	6	5′-UTR; C241T, ORF1ab; (C3037T, C14408T), Spike; (A23403G)	ORF1ab; (C8917T, G9389A), Nucleocapsid; (G28881A, G28882A, G28883C)	Spike; (T21703G, C22444T)
ILS24	6	9	ORF1ab; C8782T, Spike;G22468T, ORF8; (T28144C), Nucleocapsid; (G28878A)	Membrane; (G26730T)	ORF1ab; (C2143T, C10138T, C10702T) Nucleocapsid; (G28326T) 5′-UTR;G29742A

*Represents intergenic, non-synonymous, and synonymous mutations.

**TABLE 5:** Tabular representation of the pangolin and next strain classification of the isolates and parent strains.

Virus	Pangolin lineage	Next strain lineage
ILS01(S)	B.6.6	19A
ILS01 (A)	B.6.6	19A
ILS03_(S)	B.1	20A
ILS03_(A)	B.1	20A
ILS15_(S)	B.1.1	20B
ILS15_(A)	B.1.36.8	20A
ILS24_(S)	A	19B
ILS24_(A)	A.7	19B

### Phylogenetic Network Analysis of the Isolated Viruses

**FIGURE 7:**
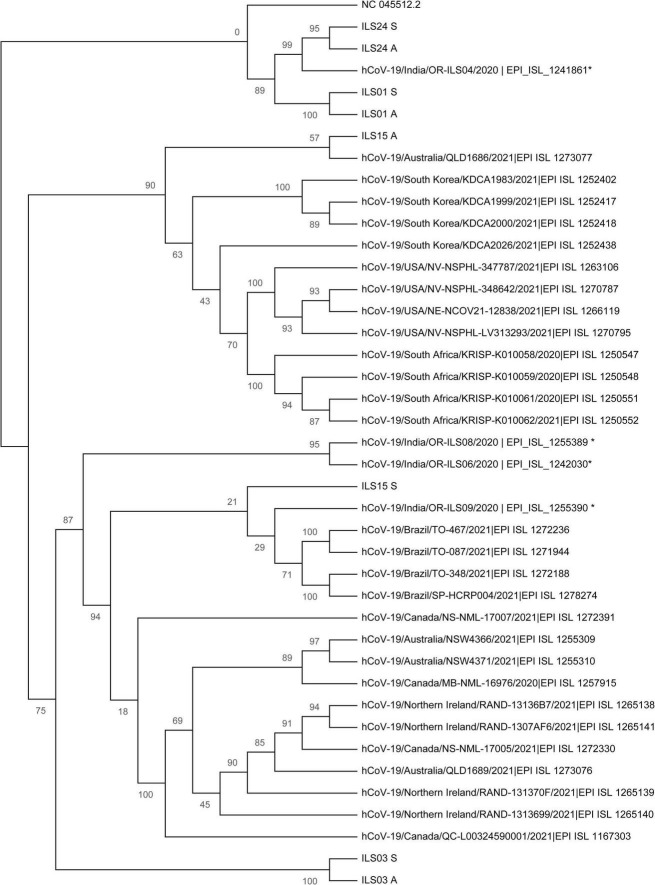
Phylogenetic network analysis of the isolated viruses: Maximum likelihood (ML) tree of studied viral sequences in combination with 33 SARS-CoV-2 genome sequences representing different countries around the globe including four sequences from Odisha, India. Bootstrap (*n* = 1,000) values are represented as branch labels.

To understand the evolution of the virus and trace the lineage, phylogenetic network analysis was performed using the genome sequence of the four isolates and 33 other largely complete sequences of the SARS-CoV-2 genome from different regions of the world. The analysis indicated that the genome sequence of the swab samples and culture adapted viruses were identical as they cluster close together in the respective clades (Figure 7), which was also in agreement with the mutational plot analysis that showed the presence of similar non-synonymous mutations throughout the respective genomes (Figure 6). Both the swab sample and adapted virus of isolates ILS01 and ILS24 clustered closely with the Wuhan reference strain, as they belong to very early clades 19A and 19B, respectively. Swab sample in the case of isolate ILS15 cluster together with viral genome isolated from India and Brazil belonging to clade 20B, whereas the adapted virus strain clustered together with the genome sequences from Australia and South Korea of clade 20A, which may be due to the five reversions found in the adapted virus. Interestingly, in the case of isolate ILS03, the swab sample and the adapted virus strain were clustered separately from the other viral genome used in this analysis.

## Discussion

In the prevailing pandemic state, it is important to isolate and characterize the disease-causing pathogen to facilitate the development of therapeutic strategies and vaccine candidates. Therefore, in this study, five circulating local strains of SARS-CoV-2 were isolated and characterized to aid in research and development, as limited COVID-19 resources were available in India. As done by other groups, Vero E6 cells were used for the isolation of SARS-CoV-2 viruses (Banerjee et al., 2020; Caccuri et al., 2020). A robust virus-induced CPE was observed from the fifth passage onward, similar to the findings of previous reports (Park et al., 2020). The viral titers were around 1 × 10^6^ TCID_50_/mL in final passages for all the isolates (Figures 1A,B), similar to the titers reported by other groups (Harcourt et al., 2020a; Brandolini et al., 2021). Subsequent infection with the isolated viruses leads to robust infection in Vero E6 cells, which was evident by an exponential increase in the viral release from 12–36 hpi and detection of infection in 80–100% of Vero E6 cells, 48 hpi. The isolates of the current study also showed infectivity in various cell lines ranging from primate to human epithelial and immune cells. The immune cells have been shown to display selective susceptibility to some viruses. For example, the THP-1 monocyte cells are not permissive to HCV and chikungunya viruses (Sourisseau et al., 2007; Revie and Salahuddin, 2014), whereas they are permissive to the dengue virus (Tsai et al., 2014). In this study, it was also found that the viral replication levels of all the isolates were similar in immune cells in comparison to the cells of epithelial lineage. Although the viral growth kinetics was alike for all the isolates, ILS03 displayed a twofold higher cytopathic effect, which might be due to the unique characteristics of ILS03 and not due to mere high viral load (Figure 1). However, further studies are warranted to characterize the mechanism specific to ILS03-mediated CPE and decipher isolate-specific variations in host–virus interactions. The current observations suggest that all five isolates belonging to the four different clades showed indistinguishable viral growth characteristics despite the genomic variations between the clades. This also indicates that the adaptive evolution occurring in the natural host may not be applicable to growth *in vitro* in cells highly permissive to viral infections.

In the natural environment, SARS-CoV-2 evolves at an estimated nucleotide substitution rate ranging between 10^–3^ and 10^–4^ substitutions per site per year (van Dorp et al., 2020), which is a very slow mutational rate. The rapid emergence of SARS-CoV-2 variants has been speculated to have happened in chronically infected immunocompromised patients with high viral replication for extended periods. Under conditions of a challenge due to transfusion of convalescent plasma or broadly neutralizing antibodies (Kemp et al., 2021), the evolution of variants has originated to evade immunity. In a natural host, due to the higher barrier toward infection, the viruses evolve and variants with higher replicative fitness get selected over time. However, in cell cultures using highly permissive cell lines, the barrier against viral replication is very low, which may not favor the rapid evolution of viral variants. The original swab samples may have population variability due to quasi-species; however, genome analysis of the swab did not reveal high population variability. Sequential passaging up to the 10th passage may have also led to a reduction in the variability of the source sample by facilitating the dominance of most fit strains. It was observed that there were a minimal number of mutations in the adapted viruses when compared to their source swab samples even after the tenth passage (Table 4 and Figure 6), suggesting that *in vitro* cultured viruses are highly stable. Funnell et al. (2021) reported mutations in the furin cleavage site (FCS) of the SARS-CoV-2 spike protein during sequential passage *in vitro*, which affected the virulence of the passaged virus. They have also reported that the observed alterations in the FCS area range from 1 to 100% upon subsequent passage of various SARS-CoV-2 viral isolates. The current viral isolates did not exhibit any mutation in the FCS region and showed mutations in the range of 0.02–0.04%, which is within the acceptable range of less than 1% mutation (Funnell et al., 2021). These isolates were also found to be highly infectious in different cell lines and in animal models of hamster and human-ACE2 transgenic mice (data not shown). The mutations were also tracked in the evolutionary server data of monkeys^1^ where they reported 213 sites with the identified features. In the current study, mutations were absent due to sequential passage in the adapted viral isolates that were similar to mutations impacted by natural evolution in the human population. The five isolates used in this study belonged to the four clades (19A, 19B, 20A, and 20B), with the clades 20A and B harboring the D614G mutation in spike protein, which has been suggested to promote the higher infectivity and transmission (Raghav et al., 2020). The observations of the current study suggest that the two vaccines, Covaxin and Covishield, are equally effective and offer protection against these viral isolates from samples collected during the first wave of COVID-19 in Odisha, India. Covaxin is a whole inactivated virus (strain NIV 2020-770), and Covishield is a chimpanzee adenovirus encoding the SARS-CoV-2 spike glycoprotein (ChAdOx1-S) based on the early viral isolates closer to the Wuhan strain. However, during the second wave, many new variants emerged across the world, and they escaped neutralization by antibodies induced by vaccines based on early isolates. The majority of the neutralizing antibodies found in convalescent sera target the spike and RBD domain of the spike (Almehdi et al., 2021; Klingler et al., 2021). Therefore, many organizations have adopted the strategy of developing vaccine candidates based on spike protein. However, further studies are warranted to evaluate the efficacy of vaccines based on whole inactivated viruses and antigenic motifs other than spike, as they can induce a broad antibody response that may be effective against the spike variants. The use of vaccine cocktails may also be an effective strategy to overcome the burden of vaccine escaping viral variants. In agreement, recent evidence suggests that a heterologous prime-boost vaccination strategy is a more effective alternative than a homologous prime-boost vaccination strategy against the emerging variants (Nordström et al., 2021).

In summary, in the current investigation, virus cultures of five SARS-CoV-2 strains belonging to various clades were established from the laboratory-confirmed SARS-CoV-2-infected patients. Their growth kinetics and genome sequences were analyzed, and hence these isolates will be highly useful resources to facilitate research and development in the field of coronavirus biology and COVID-19.

## Data Availability Statement

The datasets presented in this study can be found in online repositories. The names of the repository/repositories and accession number(s) can be found in the article/supplementary material.

## Ethics Statement

The current studies involving swab samples from the human participants were reviewed and approved by the Institutional Human Ethics Committee, Institute of Life Sciences. The Institutional Ethics Committee (IEC)/Institutional Review Board (IRB) reference number is 96/HEC/2020. The written consent form duly signed by the participants/legal guardian was taken into consideration for the concerned study. The patients/participants provided their written informed consent to participate in this study.

## Author Contributions

SC, GH, BS, SaC, and AD designed the experiments and drafted the manuscript. BS, KA, SaC, AD, SD, SK, and SG conducted the virological assays. AG and SR conducted the genomic data analysis. AS, RD, SS, TB, PP, SR, SD, RS, SoC, and AP reviewed and edited the manuscript. All authors contributed to the article and approved the submitted version.

## Conflict of Interest

The authors declare that the research was conducted in the absence of any commercial or financial relationships that could be construed as a potential conflict of interest.

## Publisher’s Note

All claims expressed in this article are solely those of the authors and do not necessarily represent those of their affiliated organizations, or those of the publisher, the editors and the reviewers. Any product that may be evaluated in this article, or claim that may be made by its manufacturer, is not guaranteed or endorsed by the publisher.

## Footnotes

1
http://covid19.datamonkey.org/
